# Reliability and longevity of implantable defibrillators

**DOI:** 10.1007/s10840-020-00920-w

**Published:** 2021-01-02

**Authors:** Robert G. Hauser, Susan A. Casey, Christopher B. Gitter, Chuen Y. Tang, Raed H. Abdelhadi, Charles C. Gornick, Larissa Stanberry, Jay D. Sengupta

**Affiliations:** 1 Minneapolis Heart Institute Foundation, 920 East 28th Street, Minneapolis, MN 55407 USA; 2 Minneapolis Heart Institute Foundation, 800 E 28th St, Minneapolis, MN 55407 USA

**Keywords:** Battery, Reliability, Longevity, Malfunction, Implantable defibrillator, Cardiac resynchronization

## Abstract

**Purpose:**

We hypothesized that data in manufacturers’ product performance reports (PPRs) can provide clinically valuable ICD and cardiac resynchronization defibrillator (CRT-D) reliability and longevity information.

**Methods:**

Data were obtained from 2019 PPRs. Kaplan-Meier (K-M) probabilities of freedom from malfunction, normal battery depletion (NBD), and NBD + malfunction were calculated for ICD and CRT-D pulse generators (PGs) with LiMnO2 or LiSVO/CFx batteries marketed in the USA from 2010 to 2019 and compared using the log-rank test. Malfunctions (MAL) included PGs that were found outside specifications.

**Results:**

Study population included 1,149,803 ICD and CRT-D PGs: Abbott (ABT; 35.1%), Biotronik (BIO; 4.6%), Boston Scientific (BSC; 23.5%), and Medtronic (MDT; 36.9%). Significant differences in reliability (*p* < 0.001), defined by freedom from MAL, were found between manufacturers; the majority of 6808 MAL occurred in ABT devices (*n* = 4045; 59.4%), followed by BSC (*n* = 2384; 35.0%), MDT (*n* = 338;5.0%), and BIO (*n* = 41; 0.6%). Battery failure (*n* = 890; 57.9%) was the most common cause of MAL compromising therapy; analysis of unique ABT battery MAL–indicated problem appeared a year prior to advisory. Significant differences (*p* < 0.001) in battery longevity, as defined by freedom from NBD, were found between manufacturers. Overall performance (freedom from NBD + MAL) favored BSC for CRT-D PGs and MDT and BIO for ICDs. BSC subcutaneous ICD reliability was inferior to its transvenous ICD (*p* < 0.001).

**Conclusion:**

PPRs contain valuable data that can be aggregated and analyzed to inform physicians. Differences in product reliability exist between manufacturers. Battery longevity has improved, but MAL have significantly impacted performance. PPR data may be useful for assessing product problems and new technology.

## Introduction

Implantable cardioverter defibrillators (ICDs) and cardiac resynchronization defibrillators (CRT-Ds) are lifesaving and life-sustaining devices that must perform reliably for years [1–5]. However, selecting the most reliable and long-lived models for implantation is an ongoing challenge for physicians [6–9]. We hypothesized that the data in the manufacturers’ regular product performance reports (PPRs) can be aggregated and analyzed to provide comparative ICD and CRT-D reliability and longevity information that is of value to clinicians who implant and follow these devices. In addition, we sought to determine if PPR data could help physicians evaluate new technologies and assess potential device performance issues.

## Methods

### Study population

The study population consisted of all Abbott, Biotronik, Boston Scientific and Medtronic, ICD, and CRT-D models that were market released in the USA from 2010 to 2019 and were powered by lithium manganese dioxide (LiMnO2) or hybrid lithium silver vanadium/carbon monofluoride (LiSVO/CFx) batteries. Excluded were older models powered by lithium silver vanadium oxide (LiSVO) batteries.

### Product performance reports

Device data were obtained from the latest editions of the online 2019 PPRs [10–13] that the manufacturers prepared according to the 2014 revised ISO standard 5841 [14]. The standard includes statistical methods for calculating PG survival probability with adjustments for underreporting. A model is included in a PPR if it is currently marketed and> 500 units have been implanted. A model is removed from a PPR if < 500 units remain in service or 20 years have elapsed since market release. Separate survival data for a given model may be reported if the clinical performance of a subset of devices is significantly different than other devices of the same model; this usually occurs as the result of a product advisory.

According to the ISO standard [14], manufacturers consider a PG to be removed for normal battery depletion (NBD) when (1) a device is returned with no associated complaint and the device has reached its elective replacement indicator(s) with an implant time that meets or exceeds the nominal (50%) predicted longevity at default (labeled) settings, or (2) a device is returned and has reached its elective replacement indicator(s) with implant time exceeding 75% of the expected longevity according to the longevity calculation tool available at the time of product introduction, and using the device’s actual use conditions and settings.

### Probability of normal battery depletion and malfunction

The Kaplan-Meier (K-M) cumulative probabilities of NBD and component malfunction were calculated by reconstructing and aggregating the K-M tables for each single-chamber (VR-ICD), dual-chamber ICD (DR-ICD), and CRT-D model listed in the manufacturer’s PPR. K-M tables were produced by using the sample sizes and cumulative survival probabilities for (1) all devices including malfunctions and NBD, and (2) for devices that were removed for malfunction only. The K-M tables for NBD were generated by censoring (subtracting) malfunctions, including battery malfunctions, that were caused by defects in design or in manufacturing.

### Malfunction

As defined in the PPRs, malfunctions included pulse generators that were returned to the manufacturer and found by analysis to have performed outside their technical specifications while implanted and in service; excluded were devices damaged after explant or caused by interaction with another device, such as lead. The manufacturers classified each malfunction as either compromising or not compromising therapy; therapy was compromised if the malfunction completely or partially jeopardized pacing or defibrillation therapy.

Abbott battery malfunctions were compared to those of Medtronic using data obtained from the manufacturers’ 2014–2019 PPRs.

### Kaplan-Meier plots

K-M plots in the figures were truncated for all manufacturers when the number of devices entering a year was less than 500. Plots differ in duration because the manufacturers introduced their LiMnO2 or LiSVO/CFx batteries at different times.

## Results

The study population consisted of 1,149,803 ICD and CRT-D pulse generators that were implanted in the USA from 2010 to 2019 (Table 1). Medtronic devices accounted for 36.9% of the population, followed by Abbott (35.1%), Boston Scientific (23.5%), and Biotronik (4.6%).

**Table 1:** Study population

Manufacturer, models, battery type (A-h capacity)	# Pulse generators (%)
CRT-D	493,478 (43)
Abbott	192,510 (39)
Quadra Assura, Unify Assura, Unify Quadra, Unify LiSVO/CFx (1.94 A-h)	
Biotronik*	13,898 (3)
Iperia 7, Ilivia 7, Intica 7, Itrevia 7, Ilesto 7 LiSVO/CFx (1.6 A-h)	
Boston Scientific	100,617 (20)
Resonate, Charisma, Momentum, Vigilant, Dynagen, Inogen, Origen, Incepta, Energen, Punctua LiMnO2 (1.9 A-h)	
Medtronic	186,453 (38)
Viva, Brava, Amplia, Claria, Compia LiSVO/CFx (1.0–1.2 A-h)	
DR-ICD	384,183 (33)
Abbott	123,370 (32)
Ellipse, Fortify LiSVO/CFx (1.75–1.94 A-h)	
Biotronik	34,528 (9)
Ilesto 7, Iforia 7, Ilivia 7, Intica 7, Itrevia 7, Iperia 7, Lumax 740, Inventra 7 DX LiSVO/CFx or LiMnO2 (1.39–1.6 A-h)	
Boston Scientific	77,170 (20)
Resonate, Charisma, Momentum, Vigilant, Dynagen, Inogen, Origin, Incepta, Energen, Punctua LiMnO2 (1.8–1.9 A-h) Dynagen, Inogen, Origen Mini LiMnO2 (1.0 A-h)	
Medtronic	149,115 (39)
Evera, Primo, Mirro LiSVO/CFx (1.0–1.2 A-h)	
VR-ICD	272,142 (24)
Abbott	87,440 (32)
Ellipse, Fortify LiSVO/CFx (1.75–1.94 A-h)	
Biotronik	4453 (2)
Ilesto 7, Itrevia 7, Lumax 740 LiSVO/CFx (1.39–1.6 A-h)	
Boston Scientific	91,949 (34)
Resonate, Charisma, Momentum, Vigilant, Dynagen, Inogen, Origen, Incepta LiMnO2 (1.8–1.9 A-h) Emblem, SQ-Rx LiMnO2 (5.67W) Dynagen, Inogen, Origin Mini LiMnO2 (1.0 A)	
Medtronic	88,400 (32)
Evera, Visia AF, Primo, Mirro LiSVO/CFx (1.0–1.2 A)	

Reported as numbers and percent

*A-h capacity*, amperes per hour; *CERT-D*, cardiac resynchronization therapy defibrillator; *DR-ICD*, dual-chamber implantable cardioverter defibrillator; *VR-ICD*, single-chamber implantable cardioverter defibrillator; *LiMnO2*, lithium manganese dioxide; *LiSVO/CFx*, lithium silver vanadium oxide/carbon monofluoride

### Reliability

**Fig. 1:**
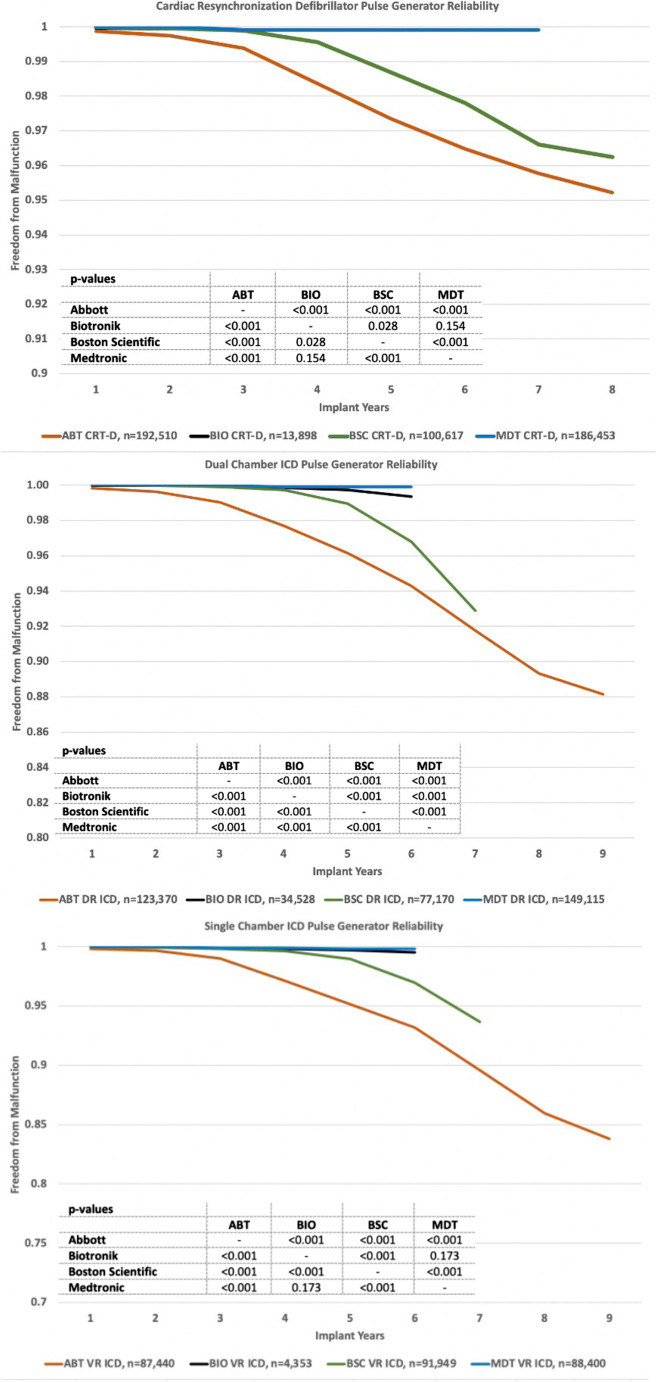
Reliability of CRT-D, DR-ICD, and VR-ICD pulse generators by manufacturer. Note: Biotronik plot is under the Medtronic plot

Reliability plots of freedom from malfunction based on K-M analysis revealed highly significant (*p* < 0.001) differences between manufacturers (Fig. 1). The majority of the 6808 tabulated malfunctions occurred in Abbott devices (*n* = 4045; 59.4%), followed by Boston Scientific (*n* = 2384; 35.0%), Medtronic (*n* = 338; 5.0%), and Biotronik (*n* = 41; 0.6%).

**Fig. 2:**
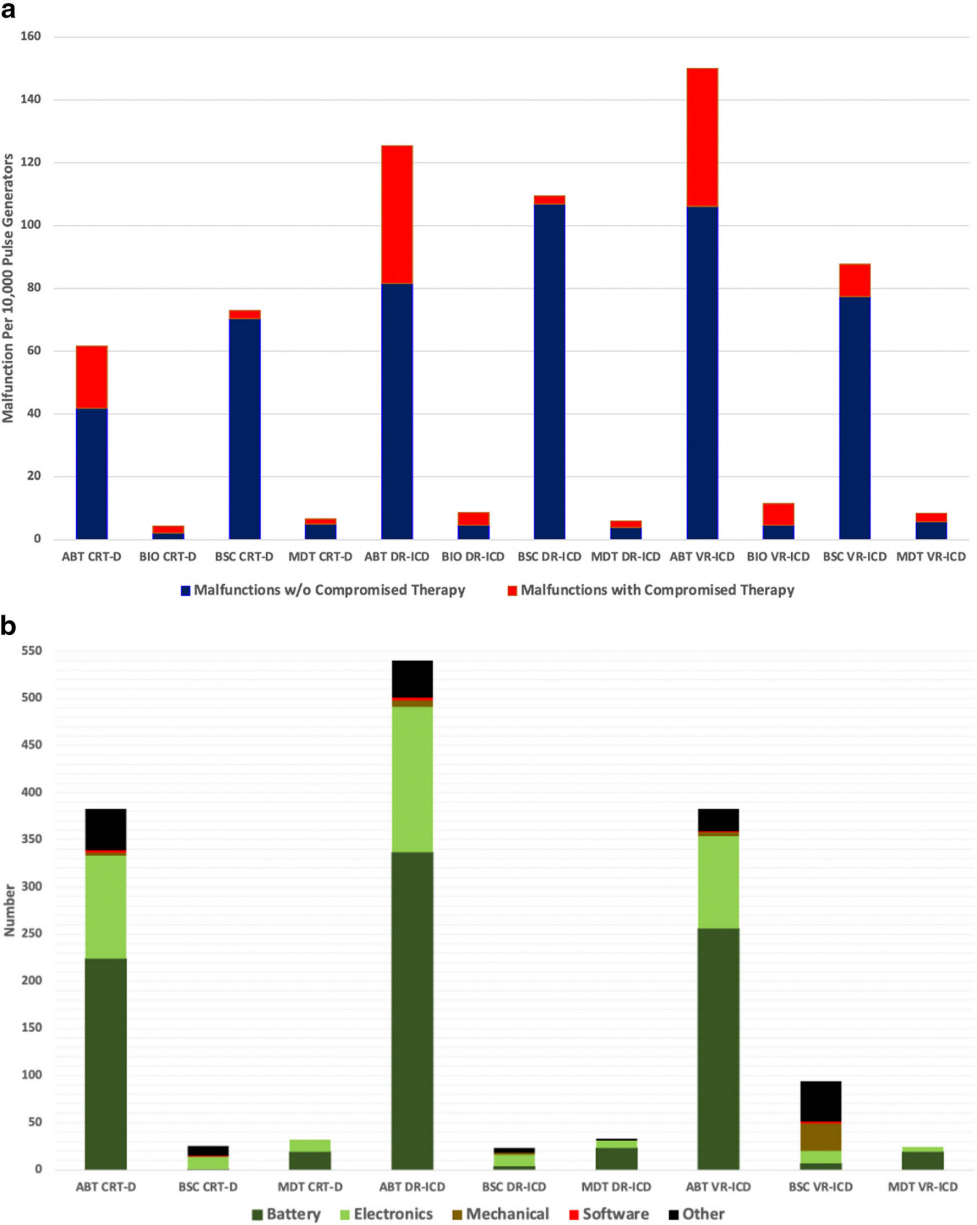
**a** Impact of malfunctions on therapy availability. **b** Causes of malfunctions that compromised therapy

As shown in Fig. 2a, Abbott device malfunctions were more likely to compromise therapy than those of other manufacturers; this was most true of Abbott’s single- and dual-chamber ICDs. The low-voltage capacitor failure caused 1972 (82.7%) of Boston Scientific’s malfunctions; less than 1% of them were classified by the manufacturer as compromising therapy.

The causes of malfunction that resulted in compromised pacing and/or defibrillation therapy are shown in Fig. 2b. Of the 1537 malfunctions, battery failure was the most common (*n* = 890;57.9%), followed by electronics (*n* = 425;27.7%), mechanical *n* = (45;2.9%), software (*n* = 10;0.7%), and other non-patterned failures (*n* = 167;1.1%).

The 3435 Abbott battery failures accounted for 50.4% of all malfunctions, and 25.0% of them compromised therapy. Except for one device, Abbott battery malfunctions occurred exclusively in models that were subject to the October 2016 product advisory [15] that alerted physicians to the possibility of rapid battery depletion due to internal short-circuiting caused by the deposition of lithium clusters. The advisory reported two patient deaths associated with this failure mode [15].

**Fig. 3:**
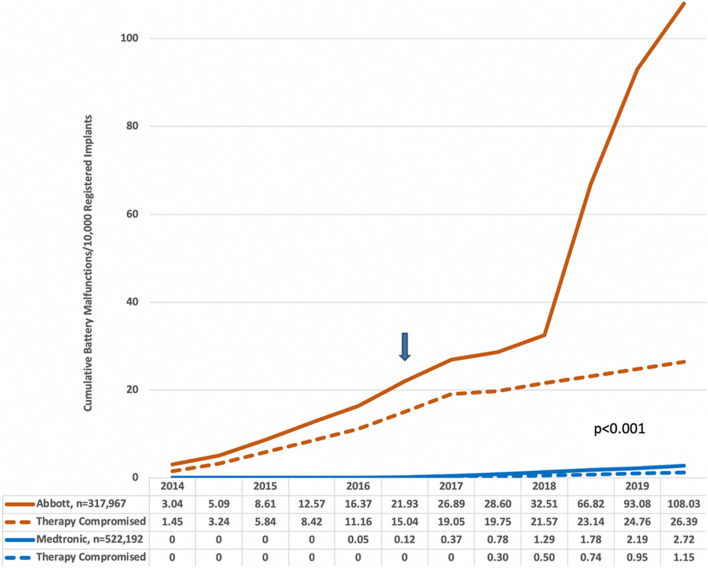
Cumulative Abbott versus Medtronic battery malfunctions. The Abbott batteries were subject to an advisory in October 2016 (arrow) that alerted physicians to the potential risk of rapid battery depletion

Figure 3 plots cumulative battery failures in Abbott models that were subject to the advisory and compares them to all Medtronic ICD and CRT-D battery malfunctions. These data, which were publicly available during the timeframes indicated, show highly significant (*p* < 0.001) differences between Abbott and Medtronic battery malfunctions beginning in 2015.

### Battery longevity

**Fig. 4:**
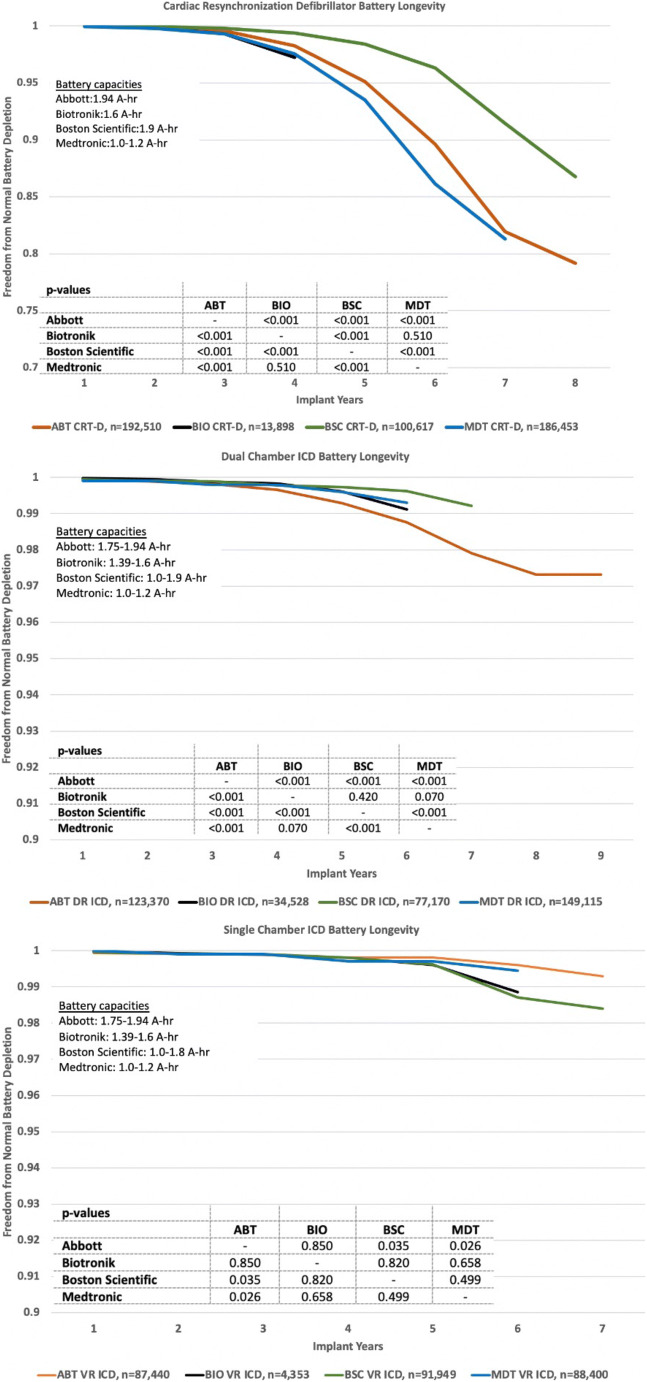
Normal battery longevity of CRT-D, DR-ICD, and VR-ICD pulse generators by the manufacturer

Longevity plots of freedom from normal battery depletion based on K-M analysis are shown in Fig. 4. The observed differences between manufacturers are highly significant (*p* < 0.001) for CRT-D and dual-chamber ICDs and less significant or insignificant for single-chamber ICDs. While Abbott and Boston Scientific pulse generators have comparable battery capacities, the former has a LiSVO/CFx battery and the later a LiMnO2 battery. All Medtronic devices have 1.0–1.2 A-hr LiSVO/CFx batteries.

### Performance

**Fig. 5:**
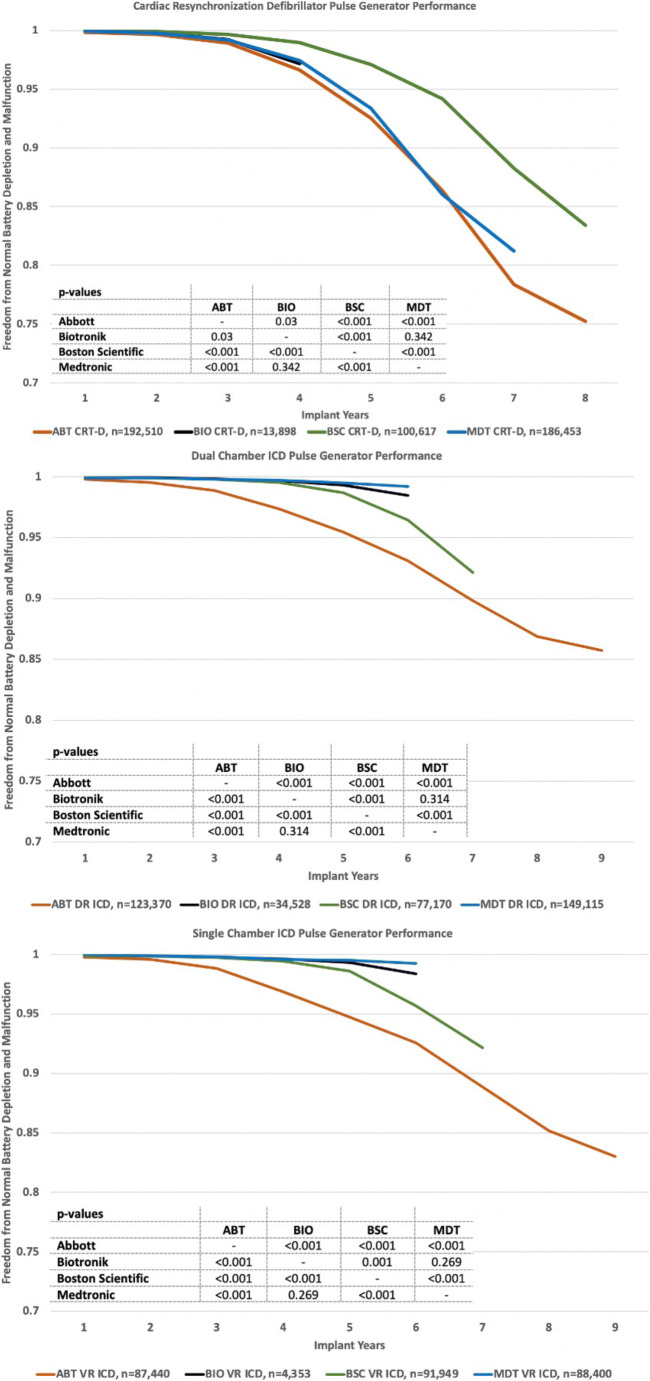
Performance of CRT-D, DR-ICD, and VR-ICD pulse generators by the manufacturer

Performance plots combining freedom from normal battery depletion and malfunction are shown in Fig. 5 for the four manufacturers’ CRT-D, DR-ICD, and VR-ICD pulse generators. Battery longevity impacted CRT-D performance more than reliability. Malfunctions so degraded single-chamber ICD performance that overall it was inferior to the performance of dual-chamber ICDs.

### Comparison of subcutaneous and transvenous ICDs

**Fig. 6:**
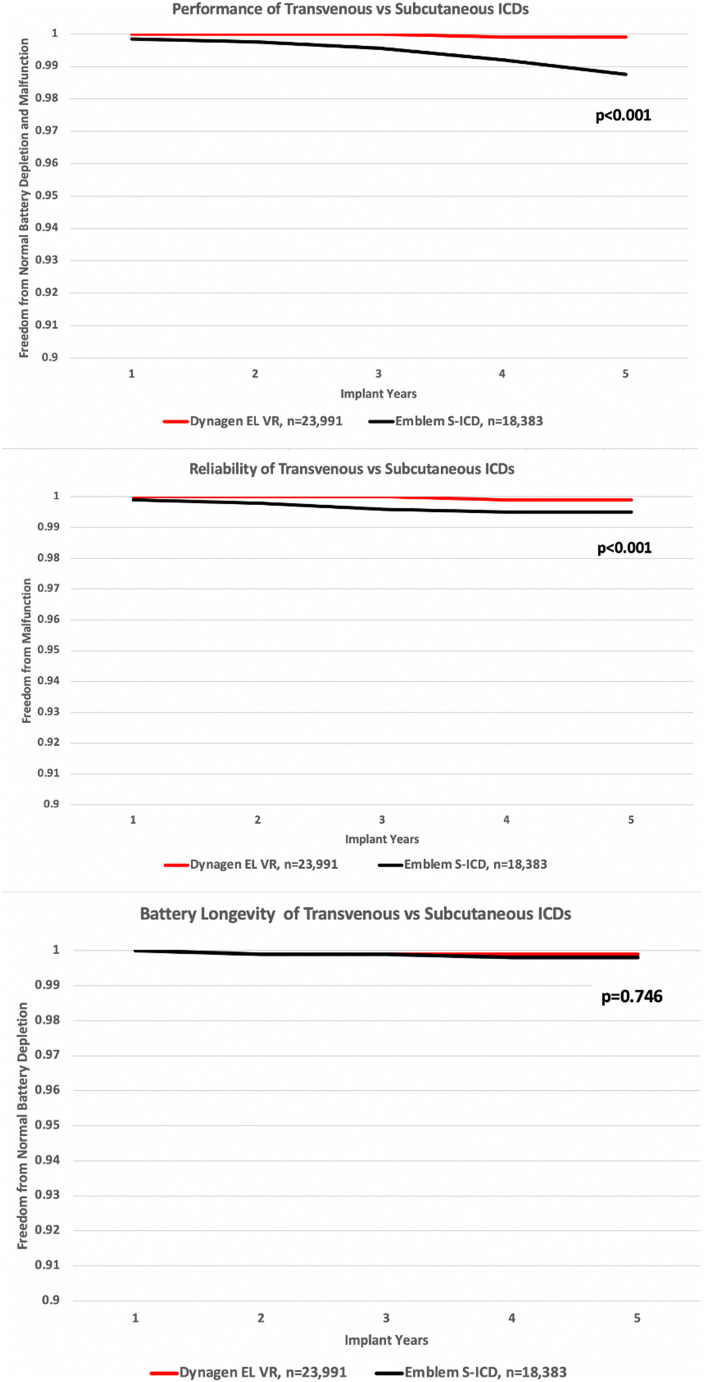
Comparison of subcutaneous and transvenous implantable cardioverter defibrillators

Figure 6 compares the performance, reliability, and battery longevity of Boston Scientific’s Emblem subcutaneous ICD (S-ICD) to its transvenous Dynagen single-chamber ICD (TV-ICD). The S-ICD is comparable to the TV-ICD in battery longevity, but its reliability is inferior; 18 of 57 (31.6%). Emblem malfunctions compromised therapy including an internal insulation issue, premature battery depletion, and diminished capacitor performance [10].

## Discussion

The results of this study show that manufacturers’ product performance reports contain data that can be aggregated, analyzed, and formatted to provide information of value to clinicians who implant and/or follow these devices. To our knowledge, this is the largest study comparing the reliability and longevity of a diverse population of implantable defibrillators, and the first to use PPR data for this purpose. We suggest that timely analyses of published PPR data can improve our understanding of implantable defibrillator performance and the capabilities of individual manufacturers. Information of this type may be useful for selecting defibrillators for implantation and for designing device follow-up protocols. In addition, we show that PPR data can be used to assess potential product problems and evaluate the performance of new technologies.

There were highly significant differences between manufacturers, and the most striking was in reliability as measured by freedom from malfunction. The Abbott LiSVO/CFx battery and Boston Scientific low-voltage capacitor failures accounted for the vast majority of malfunctions. The battery failures frequently compromised therapy and placed patients at risk for major adverse events including death [15]. Our analysis suggests that a careful examination of Abbott’s PPR data in 2014–2015 could have alerted physicians and the Food and Drug Administration (FDA) to a potentially lethal problem; furthermore, this information could have provided context for the 2014 report by Pokorny et al. [16] who described the premature failure of two Abbott Fortify ICDs at their center due to rapid battery depletion caused by lithium cluster formation near the cathode. Physicians were not notified of this battery problem until October 2016 when the incidence of battery failure that compromised therapy and caused two deaths had increased to 15/10,000 registered implants (Fig. 3).

**Fig. 7:**
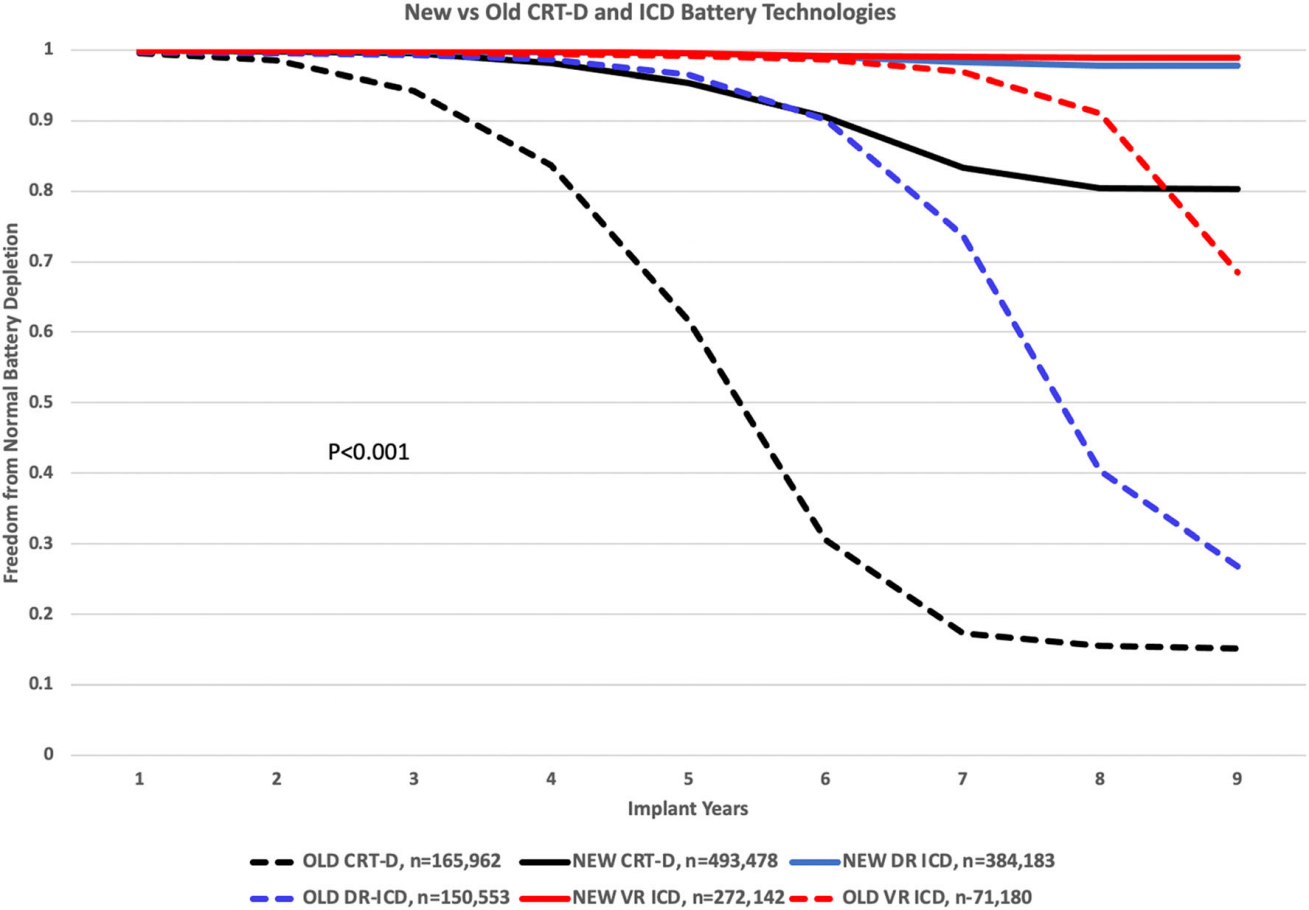
Longevity of lithium manganese dioxide and hybrid lithium silver vanadium oxide/carbon monofluoride batteries (NEW) compared to lithium silver vanadium oxide batteries (OLD). Data were obtained from 2019 Abbott, Biotronik, Boston Scientific, and Medtronic product performance reports

Compared to older lithium silver vanadium oxide batteries (LiSVO), LiMnO2 and LiSVO/CFx batteries have significantly extended the service life of implantable ICD and CRT-D pulse generators (Fig. 7). Improved pulse generator longevity reduces complications and costs and, by implication, enhances patient quality of life [17–20]. The benefits of extended battery longevity is greatest for CRT-D patients but they also apply to patients with single- and dual-chamber ICDs.

There are distinct differences in battery technology between manufacturers. Boston Scientific introduced the LiMnO2 ICD battery in 2008 [21]; it exhibits little time-dependent change in internal resistance, and thus, ICD charge times do not increase significantly until the battery reaches its recommended replacement time; this attribute may allow more efficient utilization of the battery’s capacity and thus extend its life. The LiSVO/CFx ICD battery was introduced by Abbott in 2010, and Medtronic in 2013. The high energy density of CFx provides the longevity needed for ICD and CRT-D devices, while its hybridization with silver vanadium oxide provides even higher pulse current capability than CFx alone [22]. While Medtronic’s CFx cathode consists of homogenized carbon monofluoride and silver vanadium oxide, Abbott’s battery, which is supplied by Greatbatch Medical (a subsidiary of Integer, Plymouth, MN), has a cathode composed of individual plates of carbon monofluoride and silver vanadium oxide.

For batteries of similar capacities, the Boston Scientific LiMnO2 battery longevity in our study was superior to the Abbott LiSVO/CFx battery for CRT-D and dual chamber pulse generators (Fig. 4). Both Boston Scientific LiMnO2 and Abbott LiSVO/CFx batteries had superior longevity for CRT-D applications compared to the lower capacity Medtronic LiSVO/CFx battery-powered CRT-Ds. However, the Medtronic battery outperformed Abbott’s battery in dual-chamber ICDs. Future studies should focus on the relative benefits of LiMnO2 and LiSVO/CFx battery technologies, and the impact of battery capacity on the ability of a device to match patient longevity.

Cardiac implantable electronic devices are constantly evolving, and physicians need a way to evaluate the performance of novel devices such as S-ICDs and transcatheter leadless pacemakers. Our analysis of PPR data showed that the battery longevity of the second generation Emblem S-ICD is comparable to Boston Scientific’s TV-ICDs but it appears prone to malfunctions that compromise therapy, including accelerated battery depletion [23].

This study has limitations. Battery longevity is affected by factors other than its chemistry and capacity; these include housekeeping current drain, use of advanced features, lead impedance, percent pacing, and number of shocks and chambers paced [24–26]. It is possible that these factors may have affected our battery longevity and performance data. While PPR lifetables are adjusted for underreporting, there are concerns that PPR data may not represent “real-world” experiences and may overestimate PG longevity and reliability [17,26]. The possible causes are (1) devices removed for NBD or malfunction are not returned to the manufacturer and hence are not included in survival calculations, and/or (2) the manufacturer overestimates the number of devices remaining in service and consequently inflates the number of devices at risk. When one or both occur, the failure rate is lower and the cumulative survival rate is higher than the true values. It is possible that this numerator and denominator issue caused our estimates of device performance to be overly optimistic.

### Future directions

We suggest that the information and data in future manufacturers’ product performance reports should be scrutinized by independent academic investigators for device safety signals that may indicate a potential reliability or longevity issue. Such analyses could be supplemented with information from the National Death Index and the FDA’s Manufacturer and User Facility Device Experience (MAUDE) database. The results should be shared with the manufacturer. This methodology could encourage greater transparency and a collaborative approach to improving CIED performance.

## Conclusion

Product performance reports contain valuable data that should be analyzed on a regular basis to inform physicians who implant and follow these devices. Significant differences in product reliability exist between manufacturers. While battery longevity has improved, battery and electronic reliability continue to impact CRT-D and ICD performance. Battery and electronic defects are the most common causes of malfunctions that compromise therapy. PPR data can be useful for assessing new technology or investigating a potential product problem.
